# Phenotypic Characterization and Genetic Dissection of Growth Period Traits in Soybean (*Glycine max*) Using Association Mapping

**DOI:** 10.1371/journal.pone.0158602

**Published:** 2016-07-01

**Authors:** Zhangxiong Liu, Huihui Li, Xuhong Fan, Wen Huang, Jiyu Yang, Candong Li, Zixiang Wen, Yinghui Li, Rongxia Guan, Yong Guo, Ruzhen Chang, Dechun Wang, Shuming Wang, Li-Juan Qiu

**Affiliations:** 1 National Key Facility for Gene Resources and Genetic Improvement, Key Laboratory of Crop Germplasm Utilization, Ministry of Agriculture, Institute of Crop Sciences, Chinese Academy of Agricultural Science, Beijing, China; 2 Institute of Soybean Research, Jilin Academy of Agricultural Sciences, Changchun, China; 3 Tonghua Academy of Agricultural Sciences, Meihekou, China; 4 Jilin City Academy of Agricultural Sciences, Jilin, China; 5 Jiamusi Branch of Heilongjiang Agricultural Sciences, Jiamusi, China; 6 Department of Plant, Soil and Microbial Sciences, Michigan State University, East Lansing, Michigan, United States of America; National Key Laboratory of Crop Genetic Improvement, CHINA

## Abstract

The growth period traits are important traits that affect soybean yield. The insights into the genetic basis of growth period traits can provide theoretical basis for cultivated area division, rational distribution, and molecular breeding for soybean varieties. In this study, genome-wide association analysis (GWAS) was exploited to detect the quantitative trait loci (QTL) for number of days to flowering (ETF), number of days from flowering to maturity (FTM), and number of days to maturity (ETM) using 4032 single nucleotide polymorphism (SNP) markers with 146 cultivars mainly from Northeast China. Results showed that abundant phenotypic variation was presented in the population, and variation explained by genotype, environment, and genotype by environment interaction were all significant for each trait. The whole accessions could be clearly clustered into two subpopulations based on their genetic relatedness, and accessions in the same group were almost from the same province. GWAS based on the unified mixed model identified 19 significant SNPs distributed on 11 soybean chromosomes, 12 of which can be consistently detected in both planting densities, and 5 of which were pleotropic QTL. Of 19 SNPs, 7 SNPs located in or close to the previously reported QTL or genes controlling growth period traits. The QTL identified with high resolution in this study will enrich our genomic understanding of growth period traits and could then be explored as genetic markers to be used in genomic applications in soybean breeding.

## Introduction

Soybean is a typical short-day plant [1]. Soybean cultivars adapted to specific geographical regions often differ in day-length perception that affect the length of time required to reach a given growth stage [2]. Soybean growth stages show high correlations with grain yield, plant height, pod number, and seed weight [3]. Therefore, understanding the genetic architecture of growth period traits will be useful for cultivated area division, rational distribution, and molecular breeding for soybean varieties.

Soybean was grown at a wide range of latitudes, from at least 50°N to 35°S [4], however, each soybean cultivar generally has a specific geographic or latitudinal distribution to reach its high yield. Thirteen soybean maturity groups (MG 000, MG 00, MG 0, and MG I—X) have been well developed to estimate the range of adaptability to latitudinal or geographic zones of soybean cultivars in the USA and Canada [5–6]. The growth period of soybean can be divided into vegetative growth period, from seedling to flowering, and reproductive growth period, from flowering to maturity. Hence, flowering is the transition from the two stages, which is a critical event in the life cycle of plants. Since the genes controlling soybean maturity were first reported by Woodworth (1923) [7], a series of loci (*E1* to *E9*, and J) that control flowering time and maturity of soybean were identified, and characterized at the phenotypic and genetic levels by classic methods [4, 8–15]. To date, the molecular mechanism for *E1* to *E4* loci has been uncovered [16–19], while other loci such as *E5* to *E8* remain unknown. It was reported that most of the *E* series genes had pleiotropic effects on vegetative growth period, reproductive growth period, and growth period. For example, the dominant alleles of *E2* to *E4* prolonged the reproductive period from stages R1 (days to flowering of the first flower) to R8 (full maturity) to some extent [20–21]. Relationship between maturity groups and genotypes of the *E* loci was inferred by Harosoy or Clark near isogeneic lines (NILs) [22]. Besides the cloned maturity genes, two homologs of *Flowering Locus T* (*FT*), *GmFT2a*, and *GmFT5a*, were found to encode the components of ‘florigen’, which is the mobile flowering promotion signal involved in the transition to flowering, and to coordinately control flowering in soybean [23]. Recently, the *GmFT2a* was validated to be gene *E9* [24].

For the last decades, QTL mapping based on bi-parental populations has been extensively used to dissect the genetic architecture of complex traits for soybean. From SoyBase (http://www.soybase.org/) 106 QTL related to R1, 2 QTL related to reproductive stage length (the number of days between R1 and R8), and 156 QTL related to R8, identified by bi-parental populations, could be retrieved. These QTLs were mainly distributed on the 6^th^, 7^th^, and 19^th^ chromosomes, but the physical positions and molecular functions for most of these QTL were not confirmed yet. Genome-wide association analysis (GWAS), which exploits historical recombination events, has become a powerful complementary strategy to linkage mapping for complex trait dissection at the sequence level. It has been successfully used in *Arabidopsis thaliana* [25], rice [26], maize [27], wheat [28], etc. In soybean, GWAS has been conducted for series of complex quantitative traits, such as disease resistance [29], stress tolerance [30], yield and quality related traits [31–32], and photosynthesis [33].

The aim of our study was to investigate the genetic architecture of three growth period traits, the number of days to flowering (ETF), the number of days from flowering to maturity (FTM), and the number of days to maturity (ETM) by high-throughput genetic markers in soybean. The uncovered genetic architecture will enrich our genomic understanding for growth period traits, and enhance the genetic gain to breed high yield soybean cultivars by genomic assistant breeding.

## Materials and Methods

### Plant materials and phenotypic data collection

To construct a diversity panel of phenotypes, 146 soybean accessions mainly from Northeast China were selected. The phenotypes were evaluated in two planting densities conditions, low density with one seed per 10 cm, and high density with two seeds per 10 cm. All the accessions were grown in three-row plots with 3 m in length and 0.60 m spacing between rows in a randomized blocks design with 2 replications at 4 locations, Fanjiatun experiment station of Jilin Academy of Agricultural Sciences in Jilin province from 2011 to 2015, Jilin experiment station of Jilin City Academy of Agricultural Sciences in Jilin province from 2011 to 2015, Tonghua experiment station of Tonghua Academy of Agricultural Sciences in Jilin province from 2012 to 2015, and Jiamusi experiment station of Heilongjiang Academy of Agricultural Sciences in Heilong province in 2015. The three growth period traits, the number of days to flowering (ETF), the number of days from flowering to maturity (FTM), and the number of days to maturity (ETM) were measured by the number of days from stages of plant emergence (VE) to R1, from stages of R1 to R8, and from stages of VE to R8, respectively. The criteria for the growth stages were determined following the method of Fehr and Caviness (1977) [34].

### Phenotypic data analysis

Analysis of variance (ANOVA), implemented by procedure GLM in SAS software (Release 9.1.3; SAS Institute, Cary, NC, USA), was conducted for each of the three traits by combing two planting densities, 5 years, and 4 locations. Best linear unbiased predictors (BLUPs), excluding the variations of years, locations, and experimental error, were used in the following genetic studies. The BLUPs were calculated by procedure MIXED in SAS software (Release 9.1.3; SAS Institute, Cary, NC, USA).

### DNA extraction and SNP genotyping

Genomic DNA sample were extracted from the leaf of soybean seedlings following method described by Kisha et al. (1997) [35]. All the accessions were genotyped via the Illumina SoySNP6k iSelect BeadChip (Illumina, San Diego, Calif. USA), which consisted of 5361 SNPs [36]. The chromosomal distributions, coding, and quality controlling of the SNPs were previously documented in Wen et al. (2014) [29]. By excluding the SNPs with missing rate higher than 0.25, and MAF (minor allele frequency) lower than 0.05, 4032 SNP markers were retained for the following up analysis.

### Population genetics analysis

Genetic diversity characteristics, including MAF, polymorphic information content (PIC), heterozygosity, and gene diversity were evaluated using software Powermarker V3.25 [37]. An admixture model-based clustering method was used to infer population structure and to assign 146 genotypes to subpopulations by 4032 SNP markers using the software STRUCTURE 2.3 [38]. The hypothetic number of subpopulations (*k*) was ranged from 1 to 10, and each *k* was run 10 times with a burn-in period of 100,000, and 100,000 Markov Chain Monte Carlo (MCMC) replications. The ad hoc statistics delta *k* (Δ*k*) was used to determine the number of clusters [39].

To investigate the population differentiations, analysis of molecular variance (AMOVA) [40], and *F*-statistics (*F*_*ST*_) for the inferred subpopulations were performed using Arlequin V3.11 [41]. A neighbor-joining (NJ) phylogenetic tree was constructed based on the Nei’s genetic distances matrix [37, 42] using Powermarker V3.25 [37]. Genotypic similarity among 146 accessions was evaluated by Flapjack (downloaded from https://ics.hutton.ac.uk/flapjack/), which was previously described by Milne et al. (2010) [43]. The kinship matrix was calculated using TASSEL 4.0 [44] to determine the genetic relatedness among individuals based on the sets of SNPs. The linkage disequilibrium parameter *r*^2^ to estimate the degree of LD was calculated using TASSEL 4.0 [44]. The decay distance of LD at *r*^2^ = 0.1 was assigned as the length of LD block.

### The whole—genome association analysis

GWAS was conducted for three traits related to soybean growth period by unified mixed linear model (MLM) using 4032 SNPs. Variations of population structure and kinship between accessions were both fitted into the MLM to account for multiple levels of relatedness [45]. Significant markers associated with traits were declared by Bonferroni *P*-value cut-off 0.001. Significant markers in the same LD block were viewed as one QTL region. The most significant marker in one QTL region was reported.

## Results

### Phenotypic variations, correlations, and ANOVA for growth period traits

Large phenotypic variation was observed for all the three traits of 146 accessions (Table 1). As expected, FTM was much longer than ETF, and ETM was longer than both of ETF and FTM. Thus the standard deviation (SD) of ETM was the largest, followed by FTM and ETF, while the coefficient of variation (CV) of ETF was the highest, and those of FTM and ETM were similar. Comparing the mean values of each trait under two densities, the basic statistics of each trait under two densities were fairly the same (Table 1). ETF was ranged from 31.0 d to 55.8 d with an average of 37.4 d in low planting density, and from 31.1 d to 56.7 d with an average of 37.5 d in high planting density.

**Table 1:** Descriptive statistics of best linear unbiased predictors (BLUPs) for three traits in two planting densities.

Traits^a^	Low-density					High-density				
	Min	Max	Mean	SD^b^	CV^c^	Min	Max	Mean	SD^b^	CV^c^
ETF	31.0	55.8	37.4	4.8	13.0	31.1	56.7	37.5	4.8	12.9
FTM	67.9	95.0	83.7	6.6	7.9	67.2	95.2	83.7	6.8	8.2
ETM	97.6	135.1	119.9	9.6	8.0	97.0	136.5	120.1	9.8	8.2

^a^ ETF, number of days to flowering; FTM, number of days from flowering to maturity; ETM, number of days to maturity

^b^ SD, standard deviation

^c^ CV, coefficient of variation.

**Fig 1:**
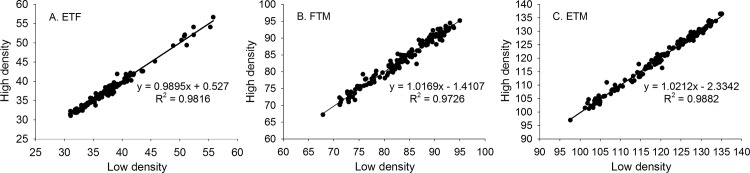
The scatter plot of the phenotype of three traits under low and high planting densities. A is for ETF, number of days to flowering; B is for FTM, number of days from flowering to maturity; and C is for ETM, number of days to maturity.

There were significant positive correlations among three growth period traits (Table 2). In low planting density, the correlation coefficients were 0.7527 for ETF and ETM, 0.8876 for FTM and ETM, and 0.3665 for ETF and FTM. In the high planting density, the correlation coefficients were 0.7645 for ETF and ETM, 0.8880 for FTM and ETM, and 0.3834 for ETF and FTM. Since ETM was derived from ETF and FTM, the correlations between ETM and ETF, and between ETM and FTM were significantly high. The low correlation between ETF and FTM indicated that ETF and FTM were independent to some extent. It was observed that the phenotypic measurements for each of the three traits under two planting densities were highly correlated, with *R*^2^ ranging from 0.97 to 0.99 (Fig 1), which explained why the values in upper triangle and low triangle of Table 2 were almost the same.

**Table 2:** Correlation coefficients between three growth period traits across the four locations.

	ETF	FTM	ETM
ETF	1	0.3665**	0.7527**
FTM	0.3834**	1	0.8876**
ETM	0.7645**	0.8880**	1

Correlation coefficients in the upper triangular are for trait performance under low density, and in the lower triangular are for trait performance under high density

** denotes the significant level under 0.01 for Pearson correlation test; ETF, number of days to flowering; FTM, number of days from flowering to maturity; and ETM, number of days to maturity.

ANOVA was conducted for each trait by combining the phenotypic data of four locations during five years for both two densities (Table 3) and for each of the density (S1 and S2 Tables). Results showed that the variation of density was significant for ETF and ETM, but not for FTM. For all the three traits, significant variations were observed among environments, replications (or blocks) within environments, genotypes, and genotype by environment (GE) interactions (Table 3). For each trait in interest, the proportions of genotypic variation were much higher than the variation of GE interaction, indicating that the genotypic variation were the major part of the phenotypic variation, which resulted in the relatively high broad sense heritabilities for three traits, i.e., 67.72% for ETF, 64.18% for FTM, and 82.37% for ETM (Table 3).

**Table 3:** Analysis of variance (ANOVA) of three traits across 15 environments and two planting densities.

Trait ^a^	Source ^b^	DF ^c^	Sum of Square	Mean Square	*F* Value	Pr > F	*H*^2^ ^d^
ETF	Density	1	67.62	67.62	14.28	0.0002	67.72
	Env	14	106893.75	7635.27	1612.07	< .0001	
	Block(Env)	15	312.88	20.86	4.40	< .0001	
	Geno	145	207550.35	1431.38	302.21	< .0001	
	Geno*Env	2030	62063.50	30.57	6.46	< .0001	
FTM	Density	1	0.93	0.93	0.08	0.7752	64.18
	Env	14	192354.13	13739.58	1205.09	< .0001	
	Block(Env)	15	1131.55	75.44	6.62	< .0001	
	Geno	145	398352.03	2747.26	240.96	< .0001	
	Geno*Env	2025	135822.74	67.07	5.88	< .0001	
ETM	Density	1	58.71	58.71	6.75	0.0094	82.37
	Env	14	111548.24	7967.73	915.99	< .0001	
	Block(Env)	15	407.50	27.17	3.12	< .0001	
	Geno	145	810759.92	5591.45	642.81	< .0001	
	Geno*Env	2025	110074.98	54.36	6.25	< .0001	

^a^ ETF, number of days to flowering; FTM, number of days from flowering to maturity; ETM, number of days to maturity

^b^ Env means environment; Block(Env) means the block nested within environments; Geno means genotype; and Geno*Env means genotype by environment interaction

^c^ degree of freedom; and

^d^ broad-sense heritability.

### Genetic diversity

**Fig 2:**
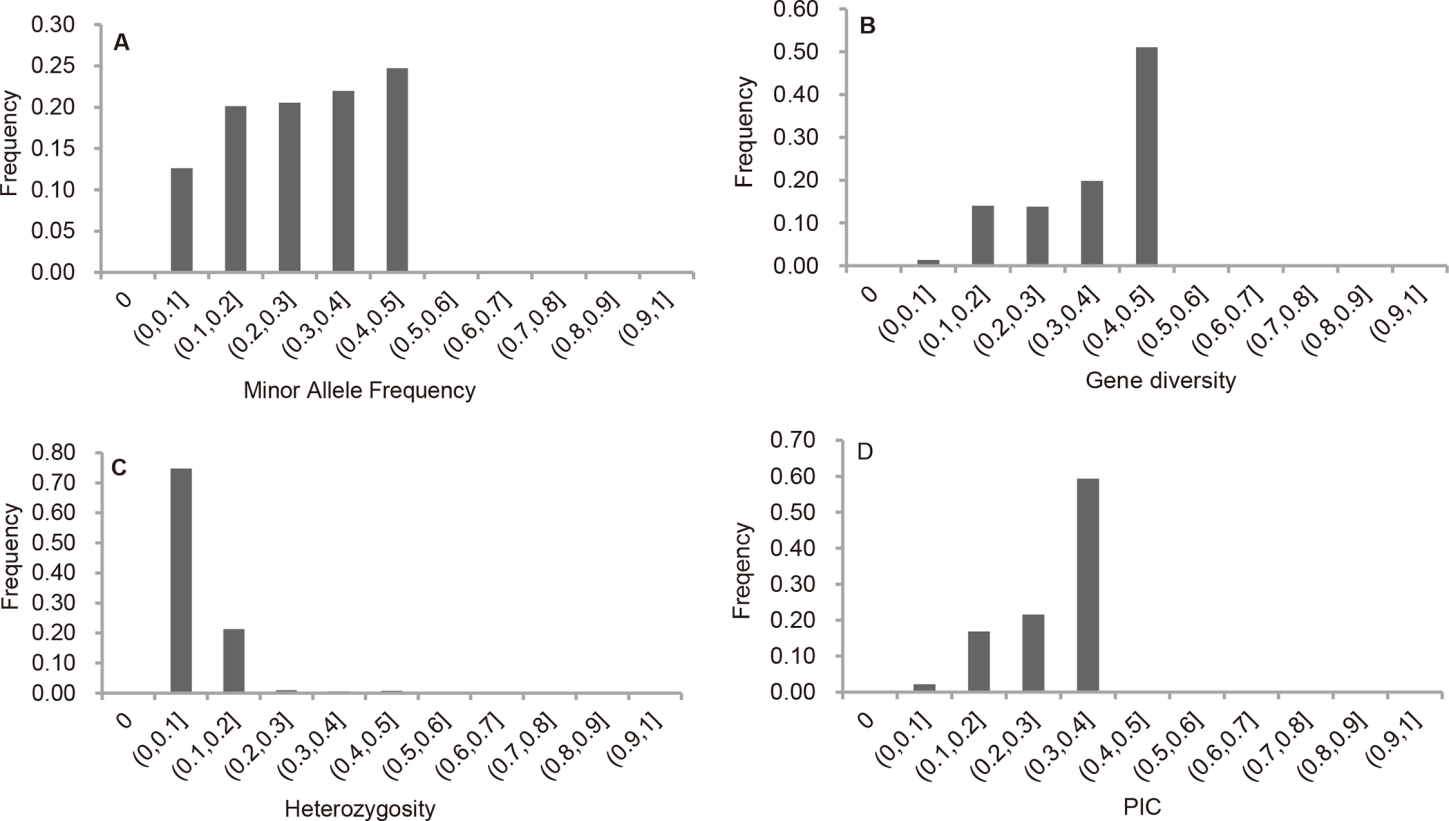
Distribution of the genetic diversity of 4,032 SNPs across 146 accessions. A is for minor allele frequency; B is for gene diversity; C is for heterozygosity; and D is for polymorphic information content (PIC).

Among the 5361 SNPs, 4032 SNPs with MAF greater than 5% were selected to estimate the genetic diversity. All the SNPs were mapped in silico and/or genetically in soybean chromosomes, and were well distributed on the 20 chromosomes [29]. The average MAF value of 4032 SNPs was 0.28, ranging from 0.05 to 0.50. About 46.7% of the SNPs had MAF greater than 0.30. The gene diversity, heterozygosity, and PIC for 4032 SNPs were 0.37, 0.09, and 0.29 on average, with ranges of 0.10–0.50, 0.00–1.00, and 0.09–0.38, respectively (Fig 2).

### Linkage disequilibrium (LD) pattern

**Fig 3:**
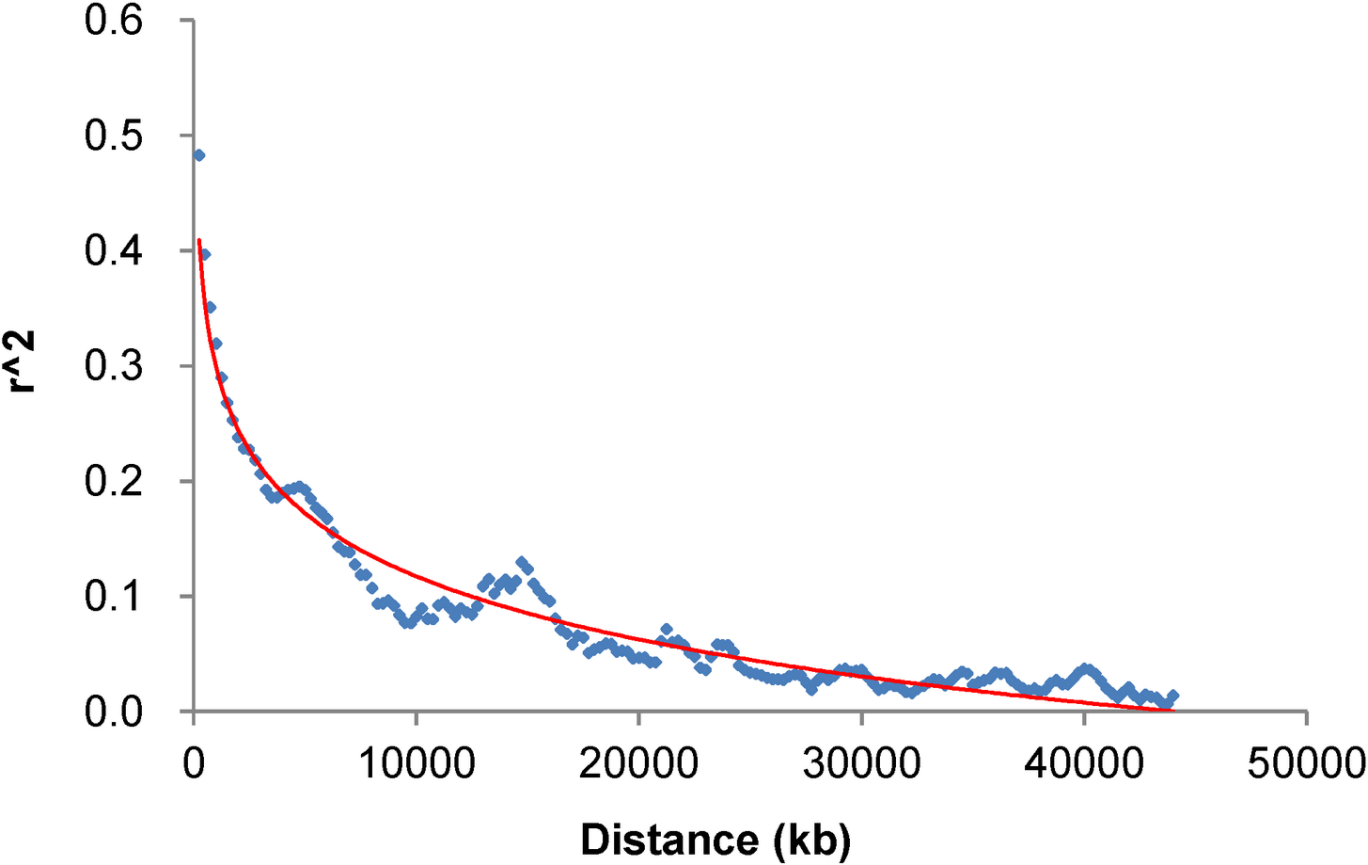
Linkage disequilibrium (LD) decay across soybean genome.

To characterize the mapping resolution of GWAS in this study, the average extent of genome-wide LD decay distance in 146 accessions with 4032 SNPs was estimated and shown in Fig 3. On average, the *r*^2^ of the whole-genome for 146 accessions was 0.23. When the physical distance was around 1,800 kb, the *r*^2^ reached to half of its maximum value 0.48. When the *r*^2^ was at 0.1, the decay distance was approximately 8,000 kb, indicating a strong LD existed in the population. Given that the average marker density was 233.53 kb in the population, the SNP markers used in this study were expected to have reasonable power to identify major QTL with large effect for three traits in 146 soybean accessions.

### Population structure

**Fig 4:**
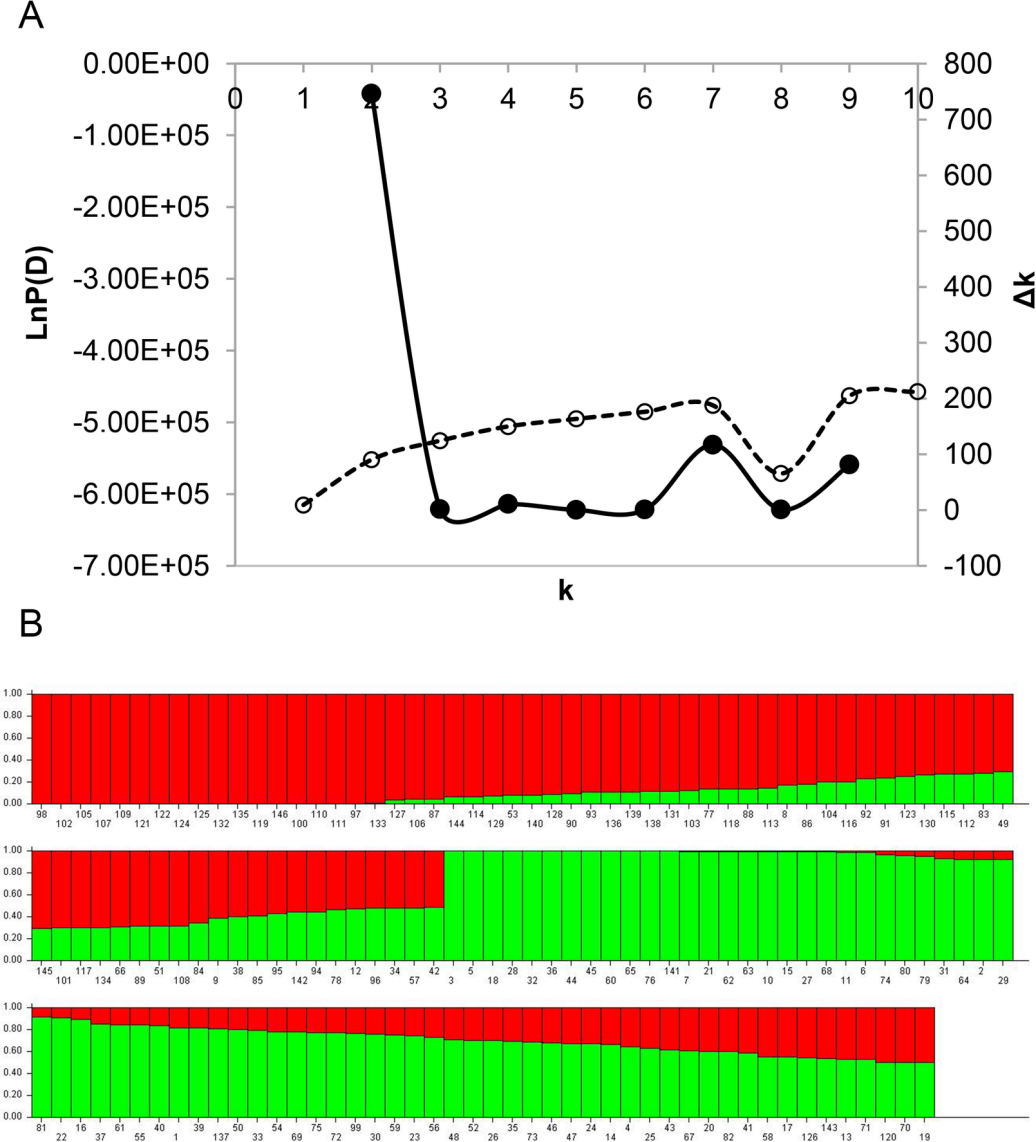
Analysis of the population structure of 146 soybean accessions. A is for the estimated Δ*k* over 10 repeats of STRUCTURE analysis; and B is for the population structure estimated by STRUCTURE. Each individual is represented by a vertical bar, partitioned into colored segments with the length of each segment representing the proportion of the individual’s genome when *k* = 2.

**Fig 5:**
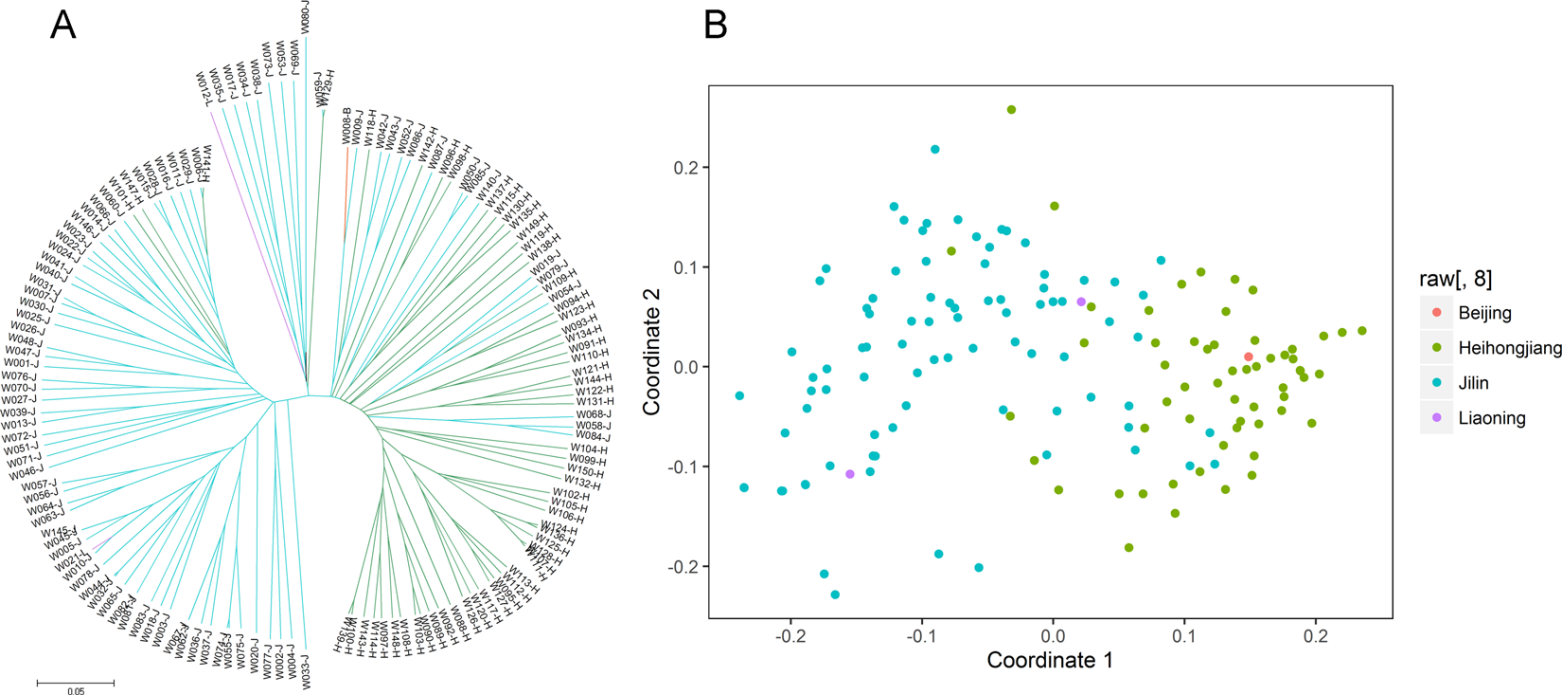
Genetic relatedness based on 4032 SNPs. A is for neighbor-joining tree; and B is for principle coordinate analysis (PCA). “W+Number” is the accession number.

To avoid false-positive associations due to population stratification, three statistical methods, including STRUCTRUE, NJ tree-based, and PCA (Figs 4 and 5) were exploited to estimate the relatedness among 146 accessions using 4032 SNPs. The distribution of LnP(D) value for each given *k* did not show a clear trend (Fig 4A). The Δ*k* reached to the highest value when *k* was at 2 (Fig 4A), which indicated that the 146 accessions could be divided into two subpopulations (Fig 4B). The measurement of population differentiation, *F*_*ST*_, was estimated at 0.18 (*P*<0.001) between the two subpopulations, suggesting high level of genetic difference (S3 Table). The result of AMOVA showed that 17.64% of the total genetic variation was among subpopulations, whereas 82.36% was within subpopulations (S3 Table).

The results of NJ phylogenetic tree and PCA (Fig 5) were consistent with the results from STRUCTRUE software (Fig 4). For subpopulation 1 (71 accessions), 53 of which originated from Heilongjiang province with relatively early maturity. The accessions in subpopulation 2 (75 accessions) were mainly from Jilin province with relatively late maturity. These results suggested that accessions from the same subgroup were closely related and generally from the same province. Such co-existence or overlapping of genetic and special differentiation is much likely due to the selection of fitness by nature and breeders. It has previously been suggested that the photoperiod response between different maturity groups may be the primary factor driving differentiation of cultivated soybean [46]. The Q matrix outputted from STRUCTURE software for two sub-populations were used to control the variation of population structure in the subsequent genetic analysis.

### QTL identified for the three traits

**Fig 6:**
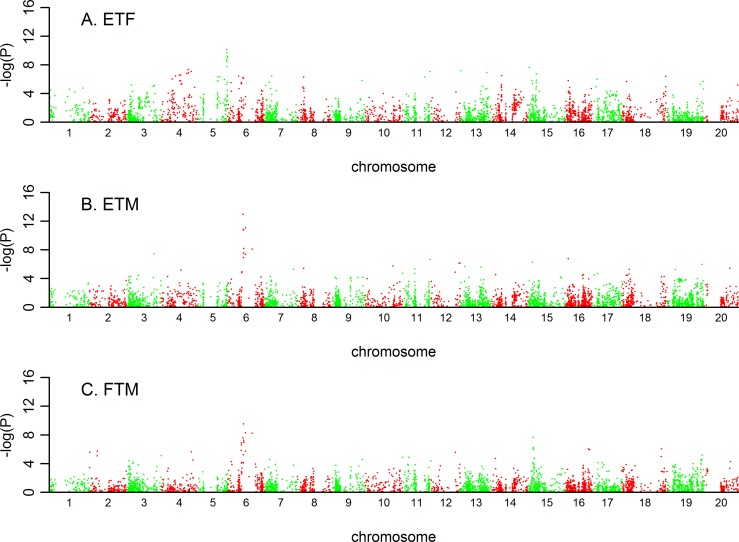
Manhattan plot from Q+K model across three traits in low density. ETF is for trait of number of days to flowering; FTM is for trait of number of days from flowering to maturity; and ETM is for trait of number of days to maturity.

**Fig 7:**
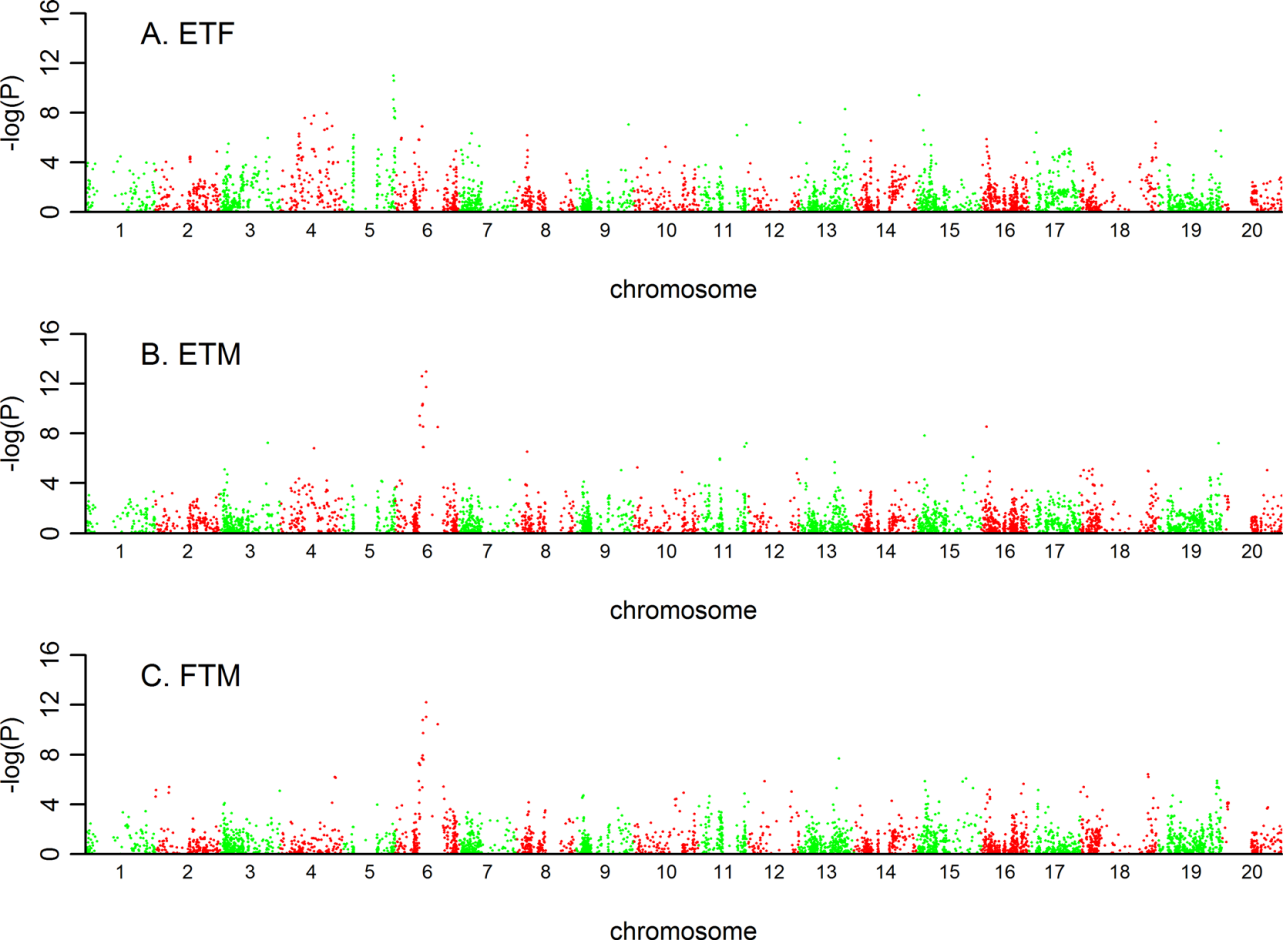
Manhattan plot from Q+K model across three traits in high density. ETF is for trait of number of days to flowering; FTM is for trait of number of days from flowering to maturity; and ETM is for trait of number of days to maturity.

**Fig 8:**
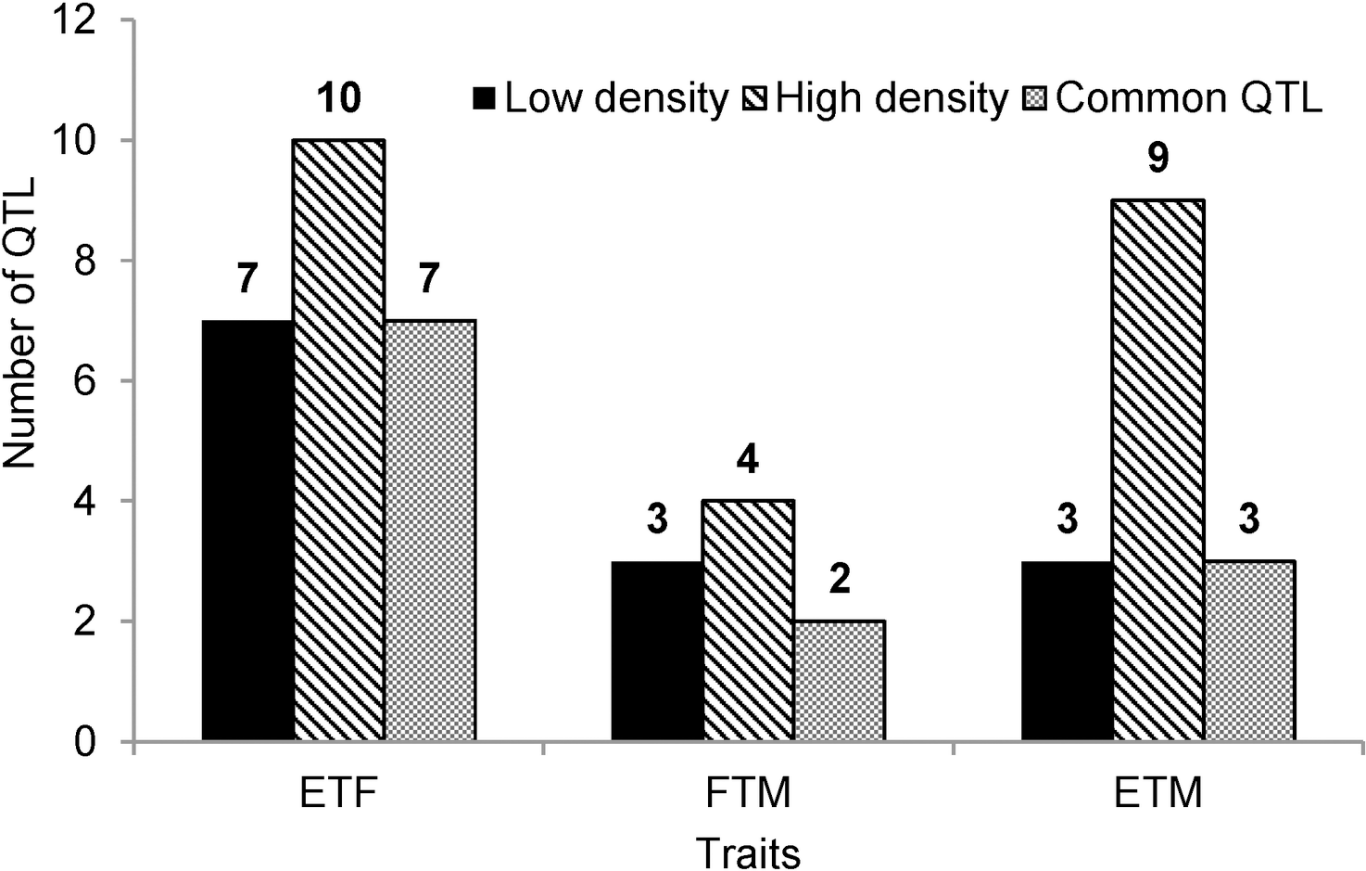
Number of QTL detected under each of low and high densities and both two densities. ETF is for trait of number of days to flowering; FTM is for trait of number of days from flowering to maturity; and ETM is for trait of number of days to maturity.

From GWAS analysis based on MLM model involved with Q matrix and Kinship matrix (S1 Fig), 19 QTL regions can be clearly identified from the Manhattan plots (Figs 6–8; Table 4). They were distributed on 11 chromosomes of soybean genome, three of each on chromosomes 3, 4, and 13, 2 on each of chromosomes 11 and 15, and one on each of chromosomes 3, 5, 9, 16, 18, and 19. The largest QTL, explaining 14.62% of the phenotypic variance, was ss245775380 on chromosome 5 associated with ETF (Table 4). Of 19 QTLs, 5 QTLs could explain more than 10% of the phenotypic variance, and the other 14 QTL could explain more than 5% of the phenotypic variance. On average, 9.53% of the phenotypic variance could be explained by each QTL.

**Table 4:** SNPs significantly associated with three traits.

Marker	Chr	Position	Associated traits (R^2^) ^a^	Reported QTLs/genes ^b^	Gene annotation ^c^
ss245178465	3	37637700	ETM (1.37), ETM (6.93)		C2H2-type zinc finger family protein
ss245412963	4	24492629	ETF (7.82)	*E8* gene [47]	PIF1-like helicase
ss245456710	4	36658492	ETF (8.77), ETF (9.56)		Unknown
ss245494974	4	40933102	ETF (7.79), ETF (7.60)	Seed weight [48]; Seed yield [49]; Pod number [50]; Seed weight per plant [51]	Unknown
ss245775380	5	40409971	ETF (12.99), ETF (14.62)	Seed weight [48, 52–53]; Seed yield [54–55]; Plant height [56]	NAD (P)-binding Rossmann-fold superfamily protein
ss245937498	6	19656740	ETM (8.69), FTM (7.66)	Pod maturity [52, 57]; Photoperiod insensitivity [58]; First flower [59]; Seed weight [48]; Seed yield [57, 60]; Plant height [57]	Unknown
ss245950346	6	21009630	ETM (14.27), FTM (11.43), ETM (14.12), FTM (12.83)	*E1* gene [17]; Seed yield [61]	A protein contains a putative bipartite nuclear localization signal and a region distantly related to B3 domain
ss245977002	6	33563355	ETM (8.67), FTM (9.00), ETM (9.27), FTM (12.04)		Plant protein of unknown function
ss247025033	9	42166200	ETF (8.24)		Prefoldin subunit; RNI-like superfamily protein; P-loop containing nucleoside triphosphate hydrolases superfamily protein
ss247557298	11	36213236	ETM (6.70)	Pod maturity [62]; Seed weight [48, 63]	Tetratricopeptide repeat (TPR)-like superfamily protein; NAD (P)-binding Rossmann-fold superfamily protein
ss247571761	11	37497232	ETF (7.89), ETF (7.95), ETM (6.89)		Uroporphyrinogen decarboxylase; Haloacid dehalogenase-like hydrolase (HAD) superfamily protein; Weak chloroplast movement under blue light
ss247852752	13	768019	ETF (7.90), ETF (7.91)		Yip1 domain; Voltage dependent anion channel; Arogenate dehydrogenase
ss248095215	13	31305831	FTM (8.25)	Pod maturity [54]; Seed fill [54]; Days to flower [64]; Days to maturity [64]; Seed weight [65–67]; Seed yield [67]; Plant height [68]; Node number [68]	Mo25 family protein; F-box/RNI-like superfamily protein
ss248139411	13	36268141	ETF (7.53), ETF (9.40)	Seed yield [69–70]	Sodium/Calcium exchanger family protein/Calcium-binding EF hand family protein; PHD finger family protein/Bromo-adjacent homology (BAH) domain-containing protein
ss248535946	15	1798512	ETF (8.75), ETF (11.43)		Protein phosphatase 2A regulatory subunit PR55; Pyridoxal phosphate phosphatase-related protein
ss248571908	15	6771127	FTM (10.14), ETM (7.73)		Disease resistance family protein/LRR family protein
ss248968508	16	4339640	ETM (8.68)	Gene *GmFT5a* [23, 71]; Reproductive stage length [72]	K+ potassium transporter
ss249946467	18	59403616	ETF (8.69)		Glycosyl hydrolase family 81 protein; Receptor-like protein kinase
ss250268653	19	47089771	ETM (7.26)	*E3* gene [18, 73]; First flower [74–76]; Flower from [75]; Seed weight [74, 77]; Seed number [74]	Pectin lyase-like superfamily protein; NAD(P)-binding Rossmann-fold superfamily protein

^a^ Traits underlined indicated that the marker-trait associations were detected under high density, otherwise under low density; ETF, number of days to flowering; FTM, number of days from flowering to maturity; and ETM, number of days to maturity

^b^ Reported QTLs/genes with italics were associated with growth period traits; and

^c^ the putative biological candidate gene in the locus or the nearest annotated gene (*Glycine max* Wm82.a1.v1) to the significant SNP.

Regarding the two planting conditions, 13 QTL were identified under low density, and 23 QTL were identified under high density. Twelve of them could be consistently identified under both densities, 7 for ETF, 2 for FTM, and 3 for ETM (Table 4; Fig 8). These results were consistent with the high correlations of phenotypic values under two densities (Fig 1).

Five QTL, three on chromosome 6 and one on each of chromosomes 11 and 15, had pleotropic effects on more than one trait. Four of them controlled ETM and FTM, and the other one controlled ETM and ETF. None of the QTL had pleotropic effects on ETF and FTM. These results were concordant with the high phenotypic correlations between ETM and FTM, and between ETM and ETF, but low correlations between ETF and FTM (Table 2). ss245950346 on chromosome 6 was consistently associated with ETM and FTM under two planting densities, explaining an average of 13.16% of the phenotypic variance. It was worth noting that ss245950346 was also in the same region with well characterized gene *E1* (Table 4). ss245937498 on chromosome 6 was identified for traits of ETM and FTM, and also reported by other literatures controlling series of traits in interest. Three pleotropic QTL, ss245977002 on chromosome 6, ss247571761 on chromosome 11, and ss248571908 on chromosome 15, had not been reported yet, but were adjacent to candidate genes with functions such as haloacid dehalogenase-like hydrolase superfamily protein, disease resistance family protein, etc.

We compared the positions of the significant SNPs identified in this study with the positions of the QTL previously reported in bi-parental mapping studies and found considerable overlap between these SNPs and the reported genes or QTL for growth period traits. Of the 19 loci, 7 were overlapped with previously reported QTL or genes related to growth period traits, and 8 loci were located in or close to the regions where QTL controlling yield or yield-related traits were reported. Sixteen QTL were closed to the candidate gene regions, and found that various types of genes were probably involving in natural variation for three growth period traits in soybean (Table 4).

## Discussions

### Large phenotypic variations and BLUP estimation

Tremendous phenotypic variations for three growth period traits can be observed in 146 soybean accessions in this study (Table 1 and S2–S4 Figs). For both densities, the deviations from the minimum values to the maximum values of ETF, FTM, and ETM were 25 days, 27 days, and 37 days, respectively (Table 1). When we dissected the phenotypic variations by ANOVA, the genotypic by environment variations were significant for all the three traits, but the genotypic variations were still the major and significant source (Table 3; S1 and S2 Tables). The broad sense heritability of ETM was the highest (82.37%), indicating that ETM performed relatively stable compared with the other two traits. To exclude the environmental variation, BLUPs per accession across environments were estimated for following up GWAS.

It is generally agreed that soybean growth period is positively correlated with yield in normal maturity conditions, and yield is significantly and positively correlated with the ratio of the reproductive period to growth period, but not with the ratio of the vegetative period to growth period. The appropriate ratio of the reproductive period to the vegetative period was supposed to be a secondary trait for indirect selection of yield and to facilitate the increasing of seed number and yield in soybean breeding [78]. In this study, ETF and ETM, FTM and ETM were significantly and positively correlated. Therefore, the affection of the ratio of the reproductive period to the vegetative period need be further investigated. The three growth period traits in this study were measured in the cultivation situation similar to that in the field, so the QTL detected in this study could be directly used in the real breeding scheme.

### SNP markers repeatedly detected or located in or near previously reported QTL

Quantitative traits were largely affected by environments. Mapping accurate and stable QTL across multiple environments was critical for molecular marker-assisted selection breeding and QTL cloning. In this study, 12 QTL were consistently identified across two planting densities (Table 4, Fig 8), which agreed with the strong correlation of trait measurements under two densities (Fig 1). In addition, 7 QTLs were located in or close to previously reported QTL or genes related to growth period. SNP ss245937498 at 19656740 bp on chromosome 6, was detected to be significantly associated with ETM and FTM, and also associated with pod maturity [52, 57], photoperiod insensitivity [58], and first flower [59]. *E1* gene was cloned by Xia et al. (2012) [17] at 20,207,279–20,207,803 bps, which is 1,827 bp away from the significant marker ss245950346 associated with ETM and FTM under two densities in this study. Significant marker ss245977002 on chromosome 6, associated with ETM and FTM, were in the downstream of *E1*. While *E7* was supposed to be either in the downstream of *E1*, or a different allele of *E1* (*Private communication*). The relationship between marker ss245977002 and *E7* need to be further investigated. Marker ss250268653 at 47,089,771 bp on chromosome 19 was associated with ETM, and was 77,821 bp away from marker satt229, which was closely linked with *E3* gene [18, 73] and controlled first flower [74–76]. On chromosome 16, ss248968508 at 4,339,640 bp was associated with ETM, and 202,142 bp far away from gene *GmFT5a* [23, 71]. In the QTL region of ss248968508, there was a QTL controlling reproductive stage length [72]. Cober et al. (2010) [47] reported that the locus *E8* was in the interval flanked by Sat_404 and Satt136 on chromosome 4. This interval is approximately 1 cM in length. Marker Satt361 located downstream of Satt136, and was approximately 0.5 cM from Satt136. SNP marker ss245412963 at 24,492,629 bp on chromosome 4 was detected to be associated with ETF in high planting density in this study, and was 378,973 bp away from Satt361. Pod maturity was similar to ETM. SNP marker ss247557298 on chromosome 11 controlling ETM was near to the marker Sat_123, which is associated with Pod maturity [62]. Marker Satt355 on chromosome 13 was reported to be associated with Pod maturity [54, 64], seed fill [54], and days to flower [64]. Marker ss248095215 used in this study controlling FTM was physically near Satt355. Therefore, some reported QTL could be validated in different environment, population, and statistical method, indicating the results in present study is rather reliable.

### SNP markers with pleiotropic effect

Of the 19 QTL detected, 5 QTL were co-association with at least two growth period traits, which coincided with significant phenotypic correlations among the traits in interest [31, 33]. In addition, it is reported that some growth period loci have pleiotropic effects on other important agronomic traits [13], such as branching [79], yield related traits [21, 80], and cleistogamy [75]. Similarly, QTL associated with yield component traits, such as seed weight, seed yield, seed weight per plant, seed number, pod number, plant height, and node number, were located in the regions where 8 QTL were identified in our study. In QTL region of ss245937498 related to ETM and FTM on chromosome 6, there were QTL not only controlling growth period traits, but also controlling seed weight [48], seed yield [57, 60], and plant height [57]. In QTL region of ss250268653 related to ETM on chromosome 19, there were QTL controlling seed weight [74, 77], seed number [74], and cleistogamous [75]. The genomic regions where multi-traits were co-associated indicated pleiotropy of single causal gene or tight linkage of multiple causal genes. In soybean molecular breeding schemes, MAS of a co-associated genetic locus could simultaneously improve multi-associated target traits, including yield and yield related traits.

### Novel SNP markers to be verified further

For the 19 QTL reported in this study, 9 of them were not reported before. One reason could be because the accessions used in this study were nearly from the maturity groups MG 000, MG 00, MG 0, and MG I, and QTL identified in accessions from other maturity groups could not present in the accessions used in this study. Soybean cultivars adapted to high latitudes have weak or no photoperiod sensitivity. Zhai et al. (2014) [81] found that e1-nf genetic groups are approximately corresponding to cultivars of MG 000, MG 00, and MG 0 groups. The second reason may be that since most of loci reported before were detected by QTL mapping based on bi-parental populations, where only alleles polymorphic between two parents were considered, thus the number of detected QTL were restricted by the allele distribution of two parents. The third possibility was that many growth period QTL have been identified in a number of different populations, however, if a QTL is controlled by a rare allele present only in a specific accession used in creating a QTL mapping population, it could not be detected in a GWAS such as reported here. The inability of GWAS to detect rare alleles occurring in one or a few members of a population under study is well documented [82–83].

Of 9 novel loci, we found that genomic regions around eight of them harbor candidate genes (Table 4). Therefore, further studies would be conducted using a large population size with more diverse genetic background, and a large number of SNPs to verify the associated markers identified in this study.

## Supporting Information

S1 Fig Kinship value between accessions among population. Each pixel in the square indicates the kinship value of corresponding individual pair as shown on the above bar.
(PDF)

S2 Fig Phenotypic distribution of ETF. Fan is for Fanjiatun experiment station; Ji is for Jilin experiment station; Tong is for Tonghua experiment station; and Jia is for Jiamushi experiment station.
(PDF)

S3 Fig Phenotypic distribution of FTM. Fan is for Fanjiatun experiment station; Ji is for Jilin experiment station; Tong is for Tonghua experiment station; and Jia is for Jiamushi experiment station.
(PDF)

S4 Fig Phenotypic distribution of ETM. Fan is for Fanjiatun experiment station; Ji is for Jilin experiment station; Tong is for Tonghua experiment station; and Jia is for Jiamushi experiment station.
(PDF)

S1 Table Analysis of variance (ANOVA) of three traits across 15 low-dense plant environments. (PDF)

S2 Table Analysis of variance (ANOVA) of three traits across 15 high-dense plant environments. (PDF)

S3 Table Analysis of molecular variance (AMOVA) and *F*_*ST*_ for two subpopulations of soybean accessions inferred from STRUCTURE. (PDF)
