# Supplementary material for: Phenotypic Characterization and Genetic Dissection of Growth Period Traits in Soybean (Glycine max) Using Association Mapping
Source: PLoS One. 2016 Jul 1;11(7):e0158602. doi: 10.1371/journal.pone.0158602 (PMC4930185; doi:10.1371/journal.pone.0158602)
Supplement: S1 Fig — Each pixel in the square indicates the kinship value of corresponding individual pair as shown on the above bar. (PDF) [file pone.0158602.s001.pdf]

S1 Fig. Kinship value between accessions among population.

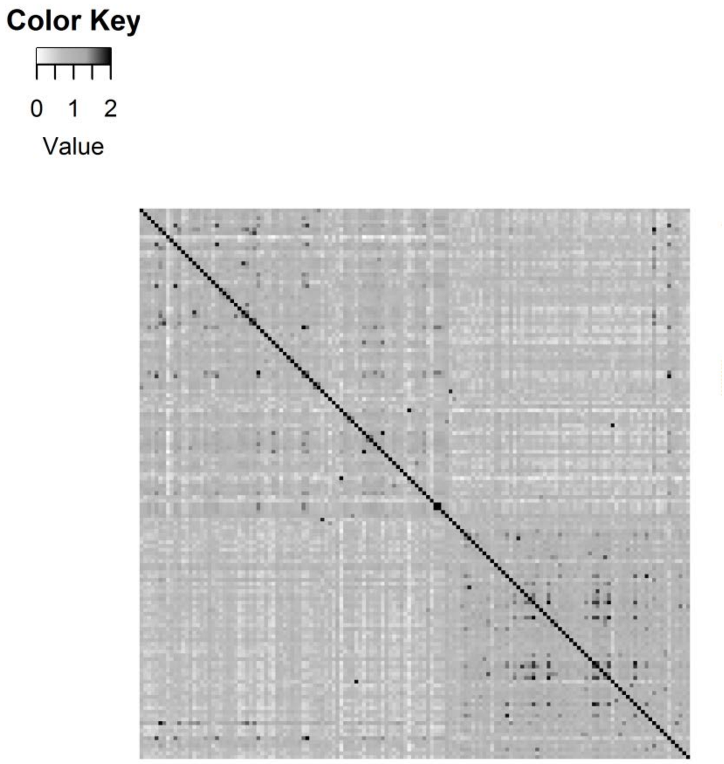

Each pixel in the square indicates the kinship value of corresponding individual pair as shown on the above bar.
