# Supplementary figures and images for: Phenotypic Characterization and Genetic Dissection of Growth Period Traits in Soybean (Glycine max) Using Association Mapping
Source: PLoS One. 2016 Jul 1;11(7):e0158602. doi: 10.1371/journal.pone.0158602 (PMC4930185; doi:10.1371/journal.pone.0158602)

25 **S2 Fig. Phenotypic distribution of ETF.**

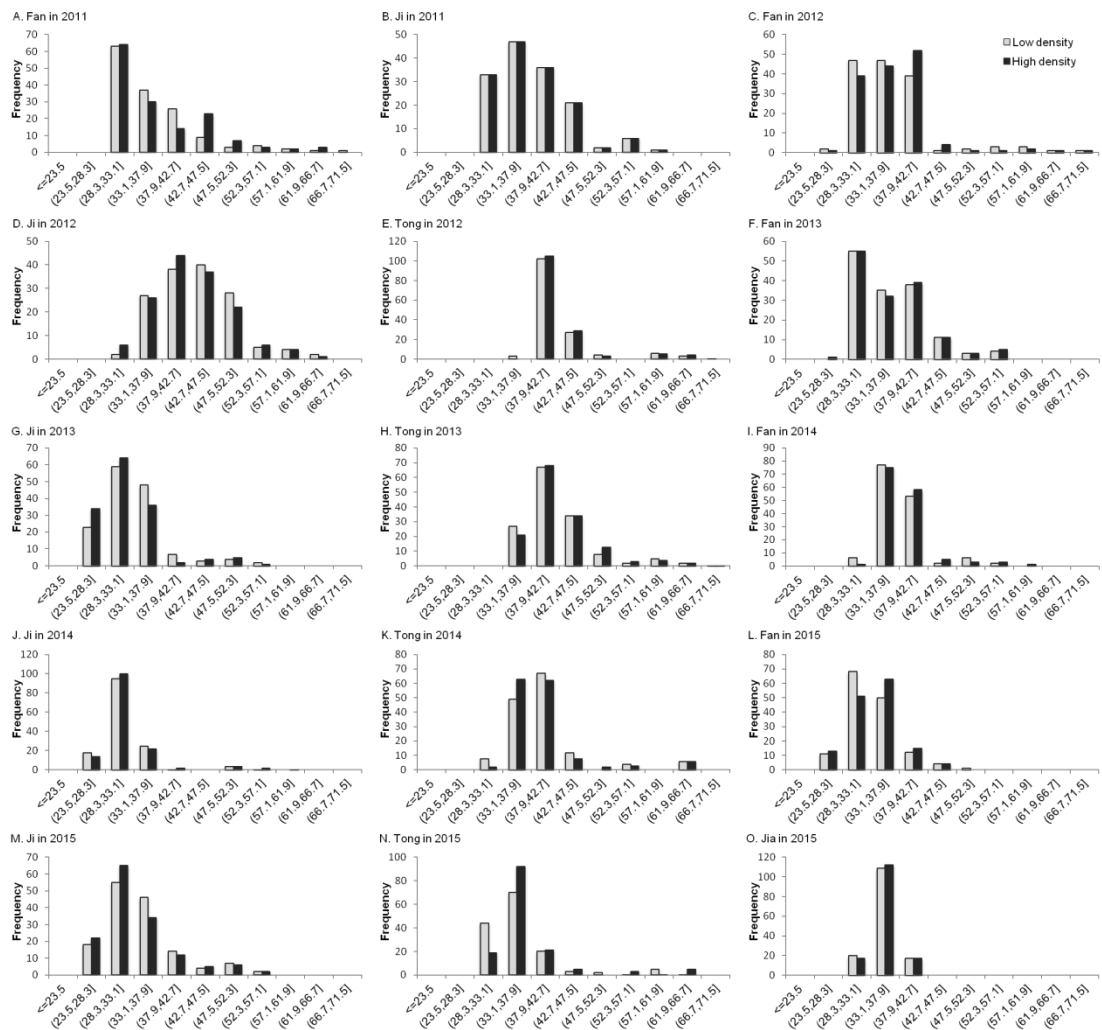

26  
27

Supplement: S2 Fig — Fan is for Fanjiatun experiment station; Ji is for Jilin experiment station; Tong is for Tonghua experiment station; and Jia is for Jiamushi experiment station. (PDF) [file pone.0158602.s002.pdf]

28 **S3 Fig. Phenotypic distribution of FTM.**

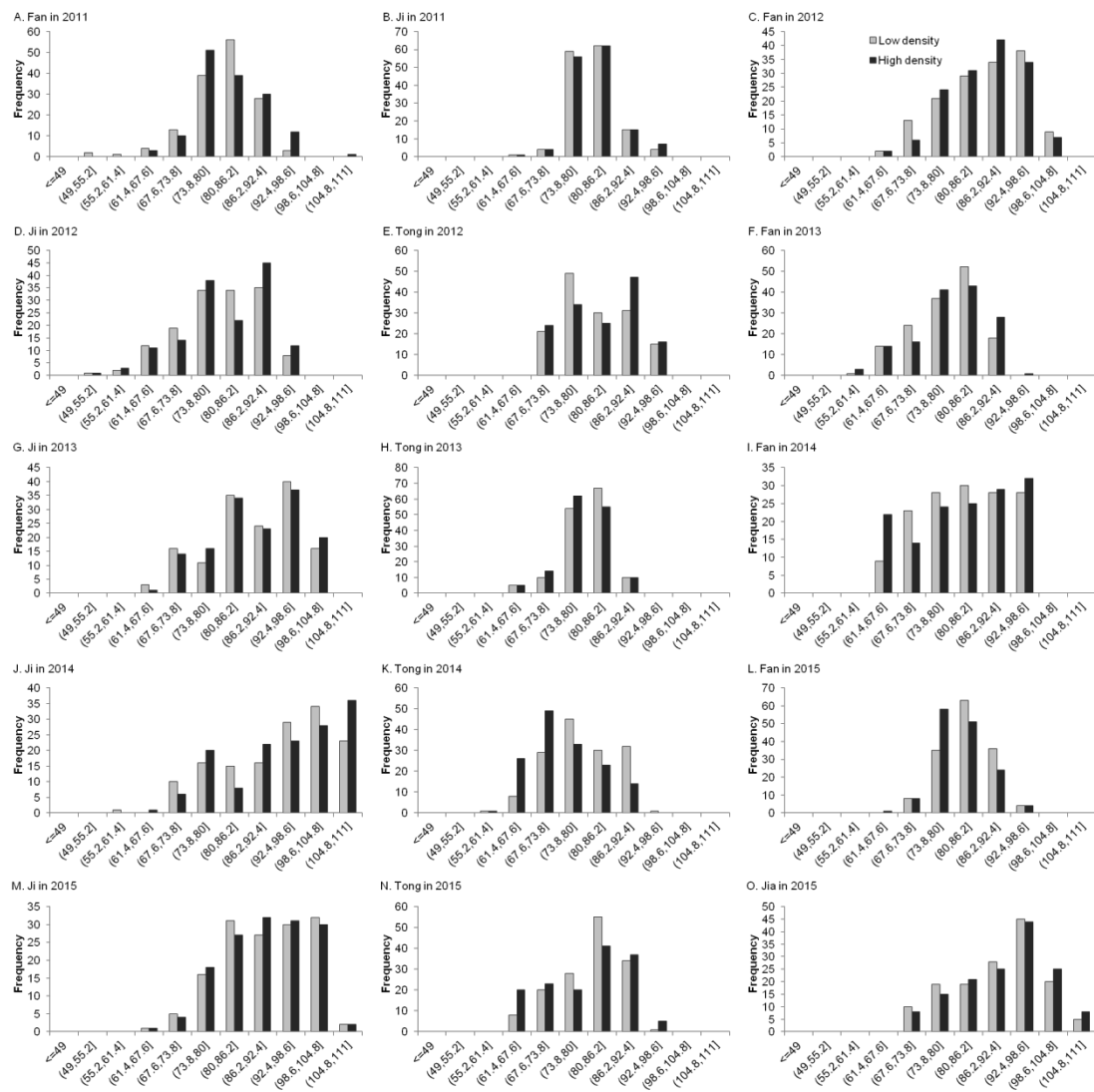

29  
30

Supplement: S3 Fig — Fan is for Fanjiatun experiment station; Ji is for Jilin experiment station; Tong is for Tonghua experiment station; and Jia is for Jiamushi experiment station. (PDF) [file pone.0158602.s003.pdf]

31 **S4 Fig. Phenotypic distribution of ETM.**

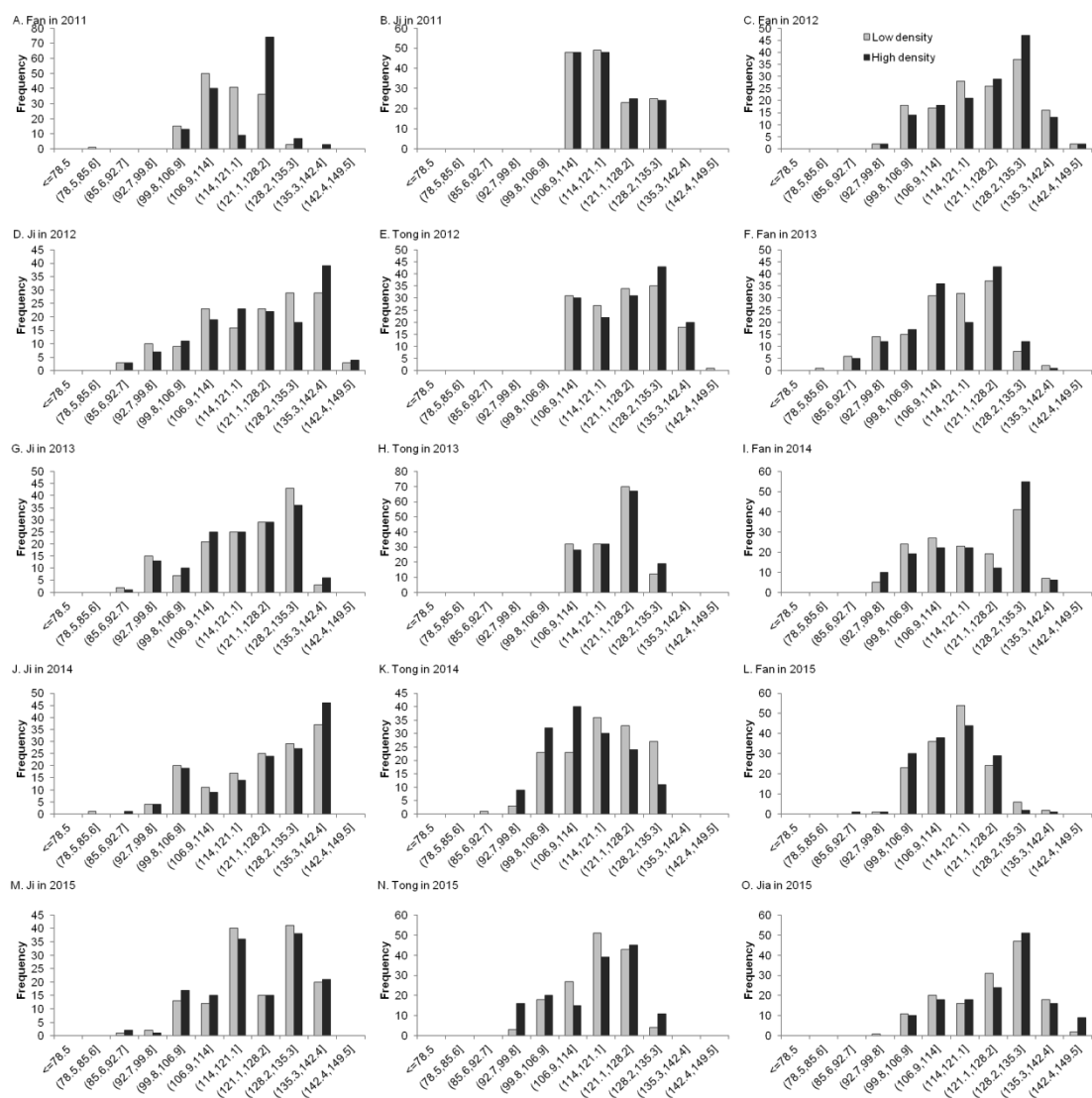

32

33

34

Supplement: S4 Fig — Fan is for Fanjiatun experiment station; Ji is for Jilin experiment station; Tong is for Tonghua experiment station; and Jia is for Jiamushi experiment station. (PDF) [file pone.0158602.s004.pdf]
