# Supplementary material for: Phenotypic Characterization and Genetic Dissection of Growth Period Traits in Soybean (Glycine max) Using Association Mapping
Source: PLoS One. 2016 Jul 1;11(7):e0158602. doi: 10.1371/journal.pone.0158602 (PMC4930185; doi:10.1371/journal.pone.0158602)
Supplement: S1 Table — (PDF) [file pone.0158602.s005.pdf]

S1 Table. Analysis of variance (ANOVA) of the three traits across 15 low-dense plant environments.

Trait [a]	Source [b]	DF [c]	Sum of Square	Mean Square	*F* Value	Pr > *F*
ETF	Env	14	51865.26	3704.66	804.36	<.0001
	Block(Env)	15	315.34	21.02	4.56	<.0001
	Geno	145	103709.42	715.24	155.29	<.0001
	Geno*Env	2030	35202.07	17.34	3.77	<.0001
FTM	Env	14	84327.65	6023.40	589.83	<.0001
	Block(Env)	15	859.31	57.29	5.61	<.0001
	Geno	145	187829.60	1295.38	126.85	<.0001
	Geno*Env	2023	80189.85	39.64	3.88	<.0001
ETM	Env	14	45721.09	3265.79	551.85	<.0001
	Block(Env)	15	195.12	13.01	2.20	0.005
	Geno	145	380540.56	2624.42	443.47	<.0001
	Geno*Env	2023	67766.03	33.50	5.66	<.0001

[a] ETF, number of days to flowering; FTM, number of days from flowering to maturity; and ETM, number of days to maturity; [b] Block(Env) means the block nested within environments; Env means environment; Geno means genotype; and Geno*Env means genotype by environment interaction; [c] degree of freedom.
