# Supplementary material for: Phenotypic Characterization and Genetic Dissection of Growth Period Traits in Soybean (Glycine max) Using Association Mapping
Source: PLoS One. 2016 Jul 1;11(7):e0158602. doi: 10.1371/journal.pone.0158602 (PMC4930185; doi:10.1371/journal.pone.0158602)
Supplement: S2 Table — (PDF) [file pone.0158602.s006.pdf]

S2 Table. Analysis of variance (ANOVA) of the three traits across 15 high-dense plant environments.

Trait [a]	Source [b]	DF [c]	Sum of Square	Mean Square	F Value	Pr > F
ETF	Env	14	54836.49	3916.89	1026.87	<.0001
	Block(Env)	15	142.42	9.49	2.49	0.0012
	Geno	145	103276.38	712.25	186.73	<.0001
	Geno*Env	2030	37853.08	18.65	4.89	<.0001
FTM	Env	14	111049.33	7932.10	1038.52	<.0001
	Block(Env)	15	813.75	54.25	7.10	<.0001
	Geno	145	207108.83	1428.34	187.01	<.0001
	Geno*Env	2025	83471.99	41.22	5.40	<.0001
ETM	Env	14	69621.33	4972.95	870.14	<.0001
	Block(Env)	15	509.80	33.99	5.95	<.0001
	Geno	145	418779.88	2888.14	505.35	<.0001
	Geno*Env	2025	65530.49	32.36	5.66	<.0001

[a] ETF, number of days to flowering; FTM, number of days from flowering to maturity; and ETM, number of days to maturity; [b] Block(Env) means the block nested within environments; Env means environment; Geno means genotype; and Geno*Env means genotype by environment interaction; [c] degree of freedom.
