# Supplementary material for: Phenotypic Characterization and Genetic Dissection of Growth Period Traits in Soybean (Glycine max) Using Association Mapping
Source: PLoS One. 2016 Jul 1;11(7):e0158602. doi: 10.1371/journal.pone.0158602 (PMC4930185; doi:10.1371/journal.pone.0158602)
Supplement: S3 Table — (PDF) [file pone.0158602.s007.pdf]

S3 Table. Analysis of molecular variance (AMOVA) and F_{ST} for two subpopulations of soybean accessions inferred from STRUCTURE.

Source of variation	Degree of freedom	Sum of squares	Variance components	Percentage of variance components
Among populations	1	14091.91	93.60	17.64
Within populations	274	126727.47	436.99	82.36
Total	275	140819.38	530.59	

Population pair-wise F_{ST}: 0.18 (P<0.001).
